# Oxygen and hydrobiological profiles of homemade manure-based tea in North Africa

**DOI:** 10.1038/s41598-025-88254-3

**Published:** 2025-02-11

**Authors:** Miliani Djezzar, Zakia Kaci, Ibrahim Yahiaoui, Crystele Leauthaud

**Affiliations:** 1 ERP Laboratory, Faculty of Nature and Life Sciences and Earth Sciences, Djilali Bounaama University of Khemis Miliana, Theniet El Had Road, 44065 Khemis-Miliana, Algeria; 2 LMVAAE Laboratory, Institute of Nature and Life Sciences, University Centre Morsli Abdellah of Tipaza, 42002 Tipaza, Algeria; 3 G-EAU, Agro Paris Tech, Cirad, IRD, INRAE, L’Institut Agro, Univ Montpellier, Montpellier, France; 4 University of California Agriculture and Natural Resources (ANR), 2801 2Nd St, Davis, CA 95618 USA; 5 Center for Agroecology, University of California, Santa Cruz, CA 95064 USA

**Keywords:** North Africa, Manure tea, Oxygen profil, Micraerobiosis, Anaerobiosis, Microbiota

## Abstract

Homemade manure tea (HMT) is commonly used in North Africa to enhance crop yields. Yet their physicochemical and biological characteristics remain poorly understood. This study evaluated oxygen and hydrobiological profiles of three types of HMT (bovine, ovine and poultry based, respectively noted HMTb, HMTo, HMTp) and compared them to control solutions of water and water supplemented with soluble NPK fertilizer. For these three types of HMT, oxygen and hydrobiological profiles were measured daily over a 7-day incubation period in three repeated, identical experiments, each comprising randomized treatments and five repetitions per treatment. Our results show that all HMT types rapidly transitioned to hypoxic conditions in the first 24h, shifting to anoxia between day 2 and day 7 depending on HMT type. This anoxic environment promoted denitrification and led to elevated NH_4_^+^ concentrations, suggesting the presence of anammox and microaerobic processes. Particulate organic matter contents and bacterial densities were highest in HMTp, while ciliate densities were highest in HMTb. These findings underscore the bioactive potential of HMT as fertilizers, with HMTp showing a favorable nitrogen profile beneficial for agricultural applications. To maintain aerobic conditions longer and reduce nitrogen losses and greenhouse gas emissions, we recommend passive or mechanical aeration, applying HMT during cooler hours, and stabilizing the pH of HMT. This study offers valuable insights to refine HMT preparation protocols, enhancing their use as bioactive fertilizers.

## Introduction

Homemade manure-based tea (HMT) is mainly traditionally produced on farms by immersing manure in water^1–5^. HMT usage is based on a more than century old crop fertilization technique whereby compounds from the substrate used (manure, compost or organic farm waste) are transferred to the crops via an aqueous solution containing organic matter, soluble nutrients, as well as many organisms, including bacteria, fungi, protozoa and nematodes^6–8^. Note that, in North Africa, using manure for organic soil amendment or preparing manure tea is actually a passive compost since, it is only applied after a decomposition phase of piled or windrowed over a period of a few weeks to more than a year^1,5,9,10^. Manure tea is a variant of compost tea that is an organic fertilizer microbial solution^11–14^. HMT can be prepared on the farm like other agricultural inputs such as homemade botanical insecticides^10,15^. Manure or organic tea is an environment friendly product that is now applied worldwide due to its natural components, simple and accessible preparation, and agronomic and environmental benefits^16–18^. HMT is especially beneficial as it may improve plant growth, particularly through better crop protection against diseases. Many studies have shown that compost tea made by soaking of compost in water for a few days, with or without forced aeration, containing organic and inorganic molecules as well as useful microorganisms and fragments and more advantageous than of compost, it in having further benefits for crop protection and growth stimulation^12,14,19^. While incubation takes place during the manure or compost maceration phase, microbiota multiply and intervene in the simplification of complex molecules to ensure their solubilization in aqueous solution^5,11,18^. Moreover, the capability of microbiota to enhance soil microbial activity has been highlighted over the last decades^11,20,21^. This practice stems from the use of agricultural by-products and residues and is sometimes geared towards safeguarding available natural resources, yet farmers mainly use it because it is inexpensive compared to other agricultural fertilizers that have to be purchased commercially^1,10,22^. HMT could supplant the intensive use of agrochemicals that may be detrimental to agroecosystem sustainability^23,24^. The hydrobiological properties of manure- or compost-based organic teas, as well as the quality and quantity of the bioactive substances they contain, are partially affected by the nature and state of decomposition of the original substrate, maceration and incubation time in water solution, aeration, and the manure/compost and water ratios^11,20,25–27^. Among these latter factors, aeration is an essential for making compost teas^28^. This is because it has a marked impact on the chemical and biological qualities of the product, thus affecting the organic matter and nutrient concentrations, but also a qualitative and quantitative impact on the microbiota components. Moreover, aerobiosis inhibits the growth of pathogenic anaerobic organisms that could develop in the tea, thereby modifying its nature and yield^5,29–32^. In North Africa, particularly in Morocco, Algeria and Tunisia, the practice of fertilizing crops with homemade manure teas of various animal origins (HMT) is widespread^1,10,33,34^, but they have yet to be qualitatively defined and controlled. In this context and in order to better define these HMT, this study aims to characterize, under small-scale on-farm preparation conditions as encountered in the Haut Cheliff region of Algeria, the oxygen profiles and hydrobiological properties of the three most commonly used HMT based on bovine (HMTb), ovine (HMTo) and poultry (HMTp) manure.

## Materials and methods

### Experimental site and homemade manure-based tea preparation

This study was conducted over a 3-month period from January to March 2020, in an open-air experimental plot at the University of Djilali Bounaama in Khemis-Miliana (Algeria). Homemade manure-based tea (HMT) was prepared by incubating the mixture of water and manure of various origins for seven days at 10% concentration, without agitation. This HMT preparation method has been previously described for manure- and compost-based teas^18,32,35–37^, and is commonly implemented in our study area (Upper Cheliff plain, Algeria). The three types of HMT used in the experiment were based on bovine (HMTb), ovine (HMTo) and poultry (HMTp) manure. The manures used to obtain HMT were processed by a traditional method whereby manure piles were left to mature for a few months under passive aeration conditions. The organic matter contents of these manures were 30.70 ± 0.23%, 31.56 ± 0.19% and 29.70 ± 0.17%, respectively.

### Experimental set-up

The experimental set-up to assess HMT was fully randomized with five replicates per treatment. The experiment was repeated once per month over three consecutive months. In addition to the three HMT treatments, two control solutions (CS) were included in the design, leading to five treatments in total. The first control was water alone (CSw), while the second was a mixture of water and soluble NPK fertilizer (15-30-15) at 1% concentration (CSf).

### Oxygen profile

HMT and CS oxygen profiles were drawn up throughout the incubation period on the basis of daily dissolved oxygen (DO) measurements performed with a WTW 3320 multiparameter meter. These measurements (expressed in mg l^−1^) were obtained by immersing the oxygen probe in the HMT and CS drums. DO levels of 2–0.5 mg l^-1^ indicated hypoxia while those < 0.5 mg l^−1^ indicated anoxia. DO levels of > 2 mg l^−1^ favored aerobiosis, while those of < 0.2 mg l^−1^ favored anaerobiosis. Levels in the 0.5–0.01 mg l^−1^ range were conducive to microaerobic activity^5,38–41^.

### Hydrobiological characterization

Hydrobiological characterization involved assessing the chemical and biological properties of HMT and CS on days 1(D_1_) and 7(D_7_) during the incubation period. Electrical conductivity (EC), total dissolved solids (TDS), pH and redox potential (ORP) were measured using a WTW 3320 multiparameter meter with independent probes. The measurements were obtained by immersing the probes in the HMT and CS drums. Ammonium, nitrite and nitrate levels were determined with a Macherey–Nagel PF-12 photometer ^*plus*^. The biological properties were defined by total bacteria, particulate organic matter (POM) and ciliated protozoa quantification. These two latter factors have not been analysed in previous studies on organic teas such as our target HMT. Yet POM functions as a carbon and nitrogen reservoir and dissolved organic matter leached from compost is generally recognized as being similar to soil borne dissolved organic matter^42–45^. Bacteria and protozoa, which interact, are also involved in POM degradation^5,45–48^. The mechanisms involved in HMT production may hence be analysed via these factors. Here, for editorial purposes, POM is considered as a biological factor despite its biochemical character and was determined by the loss on ignition method. Centrifuged HMT and CS samples were filtered on preweighed and precombusted (500 °C for 4 h) GF/F filters (Whatman GF/F, 0.7 μm). The filters with their filtrates were first dried (60 °C for 24 h) and weighed to determine the total mass. The samples were then heated at 500 °C for 4 h and weighed again for mineral residue determination. POM was determined by subtracting the mineral residue weight from the total mass^49–51^. Total bacterial abundance (expressed as CFU ml^−1^) was estimated by bacteria enumeration in all CS and HMT replicates using the spread plate method with serial dilution. Cultures were grown on nutrient agar at 28 °C incubation temperature for 48 h^41^. A standard biological and environmental analysis method was adopted for ciliate identification and enumeration. HMT and CS samples were fixed in glycerol (30%). All ciliate species were counted under a microscope using a Sedgewick Rafter counting chamber^52–55^.

### Statistical analysis

The results were expressed as means ± standard deviation (SD) for five repetitions per treatment. ANOVA was used for comparison of means using R Core Team software (2020), with the following packages: stats^56^, dplyr^57^, and Conover. Test^58^. A non-parametric Kruskal–Wallis test was applied (*p* ≤ 0.05), after confirmation of heteroscedasticity (Barlett’s test) and normality for all variables (Shapiro–Wilk test), followed by the Conover-Iman post hoc test for multiple comparisons. Linear curves represented the oxygen profiles. Histograms were drawn up to represent the POM contents, as well as bacteria and ciliate densities. A Pearson correlation matrix (r) was used to highlight correlations between POM, protozoa, bacteria and nitrogen compounds. This measures the extent of linear relationship between two variables, with values between − 1 and 1.

## Results and discussion

### Oxygen profiles

**Fig. 1:**
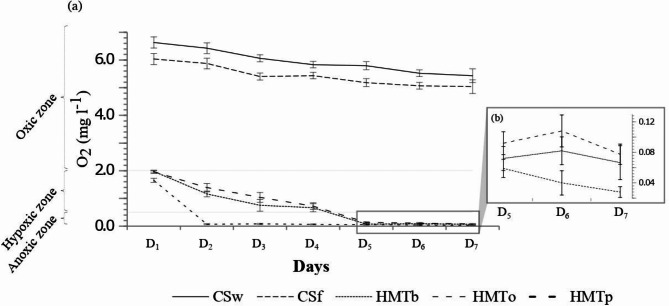
HMT and CS oxygen profiles: (**a**) throughout the 7-day incubation period; and (**b**) under anoxic conditions from D_5_ to D_7_.

The HMT and CS dissolved oxygen (DO) profiles recorded throughout the incubation period are shown in Fig. 1a. Hypoxia in HMT was observed from day 1 (D_1_), with maximum measured levels of 1.98 ± 0.03 mg l^−1^ (HMTb), 1.96 ± 0.06 mg l^−1^ (HMTo) and 1.66 ± 0.07 mg l^−1^ (HMTp), followed by anoxia on day 2 (D_2_) for HMTp (0.11 ± 0.06 mg l^−1^) and from day 4 (D_4_) for HMTb (0.67 ± 0.15 mg l^−1^) and HMTo (0.72 ± 0.12 mg l^−1^). On the last day of incubation (D_7_), and contrary to the levels recorded in CS, which ranged from 6.63 ± 0.16 to 5.04 ± 0.25 mg l^−1^, oxygen profiles of the three HMT were at their lowest level, i.e. 0.08 ± 0.02 mg l^−1^ (HMTo), 0.07 ± 0.01 mg l^−1^ (HMTb) and 0.03 ± 0.02 mg l^−1^ (HMTp). Differences between the CS and HMT samples were highly significant (< 0.0001) at each time step. Furthermore, anoxia in the poultry manure tea (HMTp) occurred quicker than in the two other teas and these differences were significant (HMTp-HMTb, *p* = 0.02; HMTp-HMTo, *p* = 0.004). The effect of the manure type and incubation period was thus clear-cut in the oxygen profiles, while the progression from hypoxia to anoxia differed in the three HMT.

Similarly, Merrill and McKeon (1998) and Francis (2016) reported that without aeration organic teas became anaerobic beyond 24–48 h incubation^20,59^. These anaerobic conditions directly related to the microbial load and food web within the manure, compost or substrate used in the making of the HMT. Despite these anaerobic conditions in the three HMT (Fig. 1a and b), the low DO levels seemed to favour microaerobiosis from D_2_ to D_7_ for HMTp (0.11 ± 0.06–0.03 ± 0.01 mg l^−1^), and from D_5_ to D_7_ for HMTb (0.07 ± 0.02–0.08 ± 0.02 mg l^−1^) and HMTo (0.08 ± 0.02–0.11 ± 0.02 mg l^−1^) (Fig. 1b). This microaerobiosis, which is linked to the presence of microaerophilic organisms and favoured by < 0.2 mg l^−1^ DO levels, has a key role in lignocellulose degradation and especially in the simultaneous nitrification and denitrification that leads to maximum nitrogen loss in the form of N_2_O^40,64–67^.

These findings suggest the importance of considering aeration or other oxygenation methods during the incubation period to prevent rapid anoxia, particularly in HMTp, which showed the fastest transition to anaerobic conditions. This could involve using aerators or manually stirring the mixture to increase oxygen levels, thereby helping to maintain aerobic conditions for a longer period. Such aeration could regulate microbial activity, slowing down oxygen depletion, which in turn helps control the denitrification process and limit methane and nitrous oxide emissions. These observations are supported by These observations are supported by previous publications^68–70^, which demonstrated that aerobic composting techniques reduce greenhouse gas emissions.

### Hydrobiological characterization

Table 1 presents the mean values obtained for pH, redox potential (ORP), conductivity (EC), TDS and nitrogen compounds (NH_4_^+^, NO_2_^-^ and NO_3_^-^) of the HMT and CS samples collected on D_1_ and D_7_. On incubation day 1 (D_1_), regarding the effects of the nature of the substrates used in HMT preparation, we noted a highly significant difference between all of the respective HMT and CS variables (*p* < 0.0001). In this comparison, the post-hoc test revealed a similarity (Table 1: D_1_ (a*), *p* = 0.123) concerning the pH of HMTb (8.12 ± 0.03) and HMTo (8.11 ± 0.02), which were slightly alkaline, contrary to HMTp and CS pH levels, which were close to neutral. Indeed, it is known that the HMT composition, as well as the quality and concentration of the associated substrates, affect the HMT chemical properties^28,71,72^. On D_1_ and in comparison, to the CSw and CSf controls, nitrogen compound (NH_4_^+^, NO_2_^−^, NO_3_^−^) dissolution was also noted on the basis of a highly significant observed difference between each of the nitrogen compounds of HMT and those of CS (*p* < 0.0001), despite the slightly nitrated nature of the CSw control solution. Otherwise, a similarity was noted between the HMTb and HMTo NH_4_^+^ and NO_2_^−^ concentrations determined in the a priori tests (Table 1: D_1_ (b*), NH_4_^+^, *p* = 0.83; (c*), NO_2_^-^, *p* = 0.7). HMTp was found to have higher NH_4_^+^ and NO_3_^-^ contents than the other HMT and CS. The different NO_3_^-^and NH_4_^+^ levels recorded on D_1_ highlighted the extent of nitrogen solubility, which is related to the manure type, maturity, age and substrate properties^35,73^. In addition, the high NO_3_^-^ concentrations and low NH_4_^+^ concentrations could be explained by the presence of nitrification, indicating that the decomposition of organic matter released into HMT was a stable process^73,74^.

**Table 1:** Physicochemical measurements on days D_1_ and D_7_.

Variables/Days		SCw	SCf	HMTb	HMTo	HMTp
O_2_ (mg l^−1^)	D_1_ D_7_	6.63 ± 0.16 5.43 ± 0.20	6.04 ± 0.20 5.04 ± 0.25	1.98 ± 0.03 0.07 ± 0.02	1.96 ± 0.06 0.08 ± 0.01	1.66 ± 0.07 0.03 ± 0.01
pH	D_1_ D_7_	7.50 ± 0.04 7.04 ± 0.09	6.90 ± 0.06 7.32 ± 0.06	a*8.12 ± 0.03 7.69 ± 0.10	a* 8.11 ± 0.02 7.72 ± 0.09	7.33 ± 0.07 7.25 ± 0.05
ORP (mV)	D_1_ D_7_	33.61 ± 0.36 15.10 ± 2.15	18.62 ± 0.37 2.65 ± 0.29	− 46.47 ± 0.38 − 87.51 ± 0.41	− 48.58 ± 0.28 − 68.37 ± 0.59	− 10.26 ± 0.50 − 11.08 ± 0.69
EC (mS cm^−1^)	D_1_ D_7_	2.73 ± 0.13 2.44 ± 0.16	10.39 ± 0.34 10.21 ± 0.17	4.34 ± 0.21 5.41 ± 0.28	4.11 ± 0.09 5.31 ± 0.12	4.65 ± 0.13 6.45 ± 0.35
TDS (mg l^−1^)	D_1_ D_7_	2.80 ± 0.13 2.51 ± 0.14	10.67 ± 0.26 10.22 ± 0.23	4.33 ± 0.27 5.41 ± 0.3	4.07 ± 0.08 3 .83 ± 0.45	4.60 ± 0.13 6.58 ± 0.30
NH_4_^+^ (mg l^−1^)	D_1_ D_7_	0.00 ± 0.00 0.97 ± 0.16	0.23 ± 0.11 2.95 ± 0.28	b*58.71 ± 2.95 379.11 ± 26.79	b*58.24 ± 4.01 413.24 ± 26.36	71.30 ± 2.59 692.51 ± 42.33
NO_2_^−^ (mg l^−1^)	D_1_ D_7_	0.06 ± 0.02 0.14 ± 0.07	0.10 ± 0.01 0.30 ± 0.01	c* 0.97 ± 0.06 0.76 ± 0.10	c* 0.97 ± 0.05 0.68 ± 0.04	1.10 ± 0.14 0.59 ± 0.05
NO3^−^ (mg l^−1^)	D_1_ D_7_	30.66 ± 5.95 27.16 ± 1.23	116.16 ± 5.77 109.20 ± 6.08	76.57 ± 2.92 140.75 ± 2.57	92.46 ± 4.59 115.69 ± 5.61	175.32 ± 8.38 218.12 ± 2.01

*: Comparison of HMTb and HMTo: (a*) = pH; NH_4_^+^ (b*) and NO_2_^-^ (c*). (D_1,_ pH,a*: *p* = 0.123; NH_4_^+^, b*: *p* = 0.83; c*: *p* = 0.7).

The significant difference in the variation patterns of some HMT and CS chemical properties between D_1_ and D_7_ (*p* < 0.0001) also reflected the effects of incubation time. Based on these patterns, we noted (Table 1): i) a pH shift towards acidity, except for the CSf pH which tended to shift towards alkalinity (7.32 ± 0.06). This pH shift towards acidity in HMT could be explained by the anaerobic conditions, whereby carbonic acid from the CO_2_ produced by microbial activity and the resulting decay affect the NH_4_^+^/NH_3_ balance^75,76^. This decrease in pH was also in line with the marked NH_4_^+^ increase noted at D_7_ in the three HMT, especially in the HMTp samples (Table 1). This trend was linked to the hypoxia- and anoxia-induced denitrification process that we observed throughout the incubation period. Hence, considering the dominant presence of NO_3_^-^ and NH_4_^+^ between D_1_ and D_7_ (Table 1), we noted that contrary to the two CS that were characterized by low N compound dynamics the denitrification process was under way as early as D_1_ and was ongoing at D_7_ in the three HMT, thereby giving rise to significant NH_4_^+^ production that could only be related to the initial NO_3_^-^ content in HMT. The low of NO_2_^−^ concentrations measured at D_1_ and D_7_ in the three HMT (Table 1) indicated that there had been no accumulation of this compound while also showing that the denitrification process was still under way. Note that in aqueous media this denitrification process is inhibited when the pH is in the 6.5–7.0 range^77^, but this was not the case for the pH in the three HMT, which ranged from pH 7–8 (Table 1). This trend was also confirmed by the oxidation–reduction (or ‘redox’) potential (ORP) findings, which indicated despite its attenuation due to incubation (in oxidizing-reducing terms) that the CS were in the oxidation process, contrary to the HMT which were undergoing reduction (Table 1). As noted by some authors, redox and acid–base reactions are essential for the maintenance of all biological activities. However, unlike pH, which is considered a key variable, agronomists focus very little on ORP, which explains why no data on HMT antioxidant properties are available in the literature^3,78^. In other disciplines, and in contrast to pH and DO, ORP is considered to be one of the most consistent and sensitive indicators of small dissolved oxygen changes, due to: its high signal reflecting the presence of nitrification, denitrification, ammonium and oxygen concentrations, the anammox process that promotes NH_4_^+^oxidation via the use of nitrite as electron acceptor and CO_2_ as a carbon source to produce N_2_ gas under anaerobic conditions, organic matter concentration and substrate depletion^79–84^. Based on research findings on ORP in aqueous media focused on farm waste leachates and wastewater under fermentation conditions, this parameter can be used as an indicator to monitor the risk of nitrogen loss through denitrification, microaerobiosis or anammox^81,85^. Under anaerobic conditions corresponding to a denitrification phase, an ORP in the − 50–50 mV range is conducive to anammox, a process in which NH_4_^+^ oxidation leads to gaseous N_2_ production^81,86^. Dissolved O_2_ levels > 0.5 mg l^−1^ are thus inhibitory for anammox bacteria, whereas denitrification exhaustion may occur with an ORP of around − 150 mV^79,87^. Regarding the latter ORP, pH and dissolved O_2_ data, our results highlighted possible nitrogen loss in the three HMT, but to varying degrees and via different mechanisms (Table 1). For HMTp, which was characterized by a stable pH (D_1_: 7.33 ± 0.07; D_7_: 7.25 ± 0.05), ORP (D_1_: 10.26 ± 0.50 mV; D_7_: − 11.08 ± 0.69 mV) and < 0.5 mg l^−1^ dissolved O_2_ content (D_2_: 0.07 ± 0.02 mg l^−1^ D_7_: 0.03 ± 0.01 mg l^−1^), the nitrogen loss could have been promoted by the anammoxic pathway throughout the incubation period. Applying HMTp at cooler times of the day, such as early morning or evening, could further limit nitrogen loss and improve nitrogen retention for crops. Additionally, diluting HMTp in water could help reduce NH_4_^+^ and NO_3_^−^ concentrations, tailoring nutrient levels to meet the needs of crops and soils with lower nitrogen demands^88–91^. For HMTb and HMTo, nitrogen loss could have occurred via microaerobiosis from D_4_ onwards, as discussed with regard to the oxygen profile (Fig. 1a and b), due to the < − 50 mV ORP levels for HMTb and HMTo (Table 1) between D_1_ and D_7_ and the > 0.5 mg l^−1^ DO levels from D_1_ to D_4_. Such insights could lead to more effective fertilizer timing and management practices to minimize nitrogen volatilization and maximize its availability in the soil. This nitrogen loss which could have been induced by volatilization, anammox reaction or microaerobiosis has not been documented in the scientific literature describing HMT production processes geared towards crop fertilization. Note that, although we did not measure the extent of N loss here, it was barely noticeable in the physicochemical variation patterns of the three HMT over the 7-day incubation period, due to the high stock and availability of NO_3_^−^, which was continuously supplied to the solution by the manure. To maximize nitrogen retention, it is crucial to maintain stable pH levels during incubation. This can be achieved by controlling anaerobic conditions to reduce pH drops from CO_2_ production, adjusting incubation times appropriately, and applying controlled aeration if possible to prevent CO_2_ accumulation^74–76^. The incubation effects from D_1_ to D_7_ were also observed when comparing the HMT and CS conductivity and TDS levels at D_1_ and those at D_7_ (Table 1; *p* < 0.0001). The significant differences in EC and TDS between the three HMT at D_1_ and D_7_ (*p* < 0.0001) were related to the manure/water volume ratio, as well as the manure substrate nature, particle size distribution, incubation time and water-soluble component concentrations^26,92–94^. This suggests that the higher conductivity values noted after incubation in HMTp, and to a lesser extent in HMTb and HMTo (Table 1), were influenced by the release of soluble salts such as ammonium^93^.

**Fig. 2:**
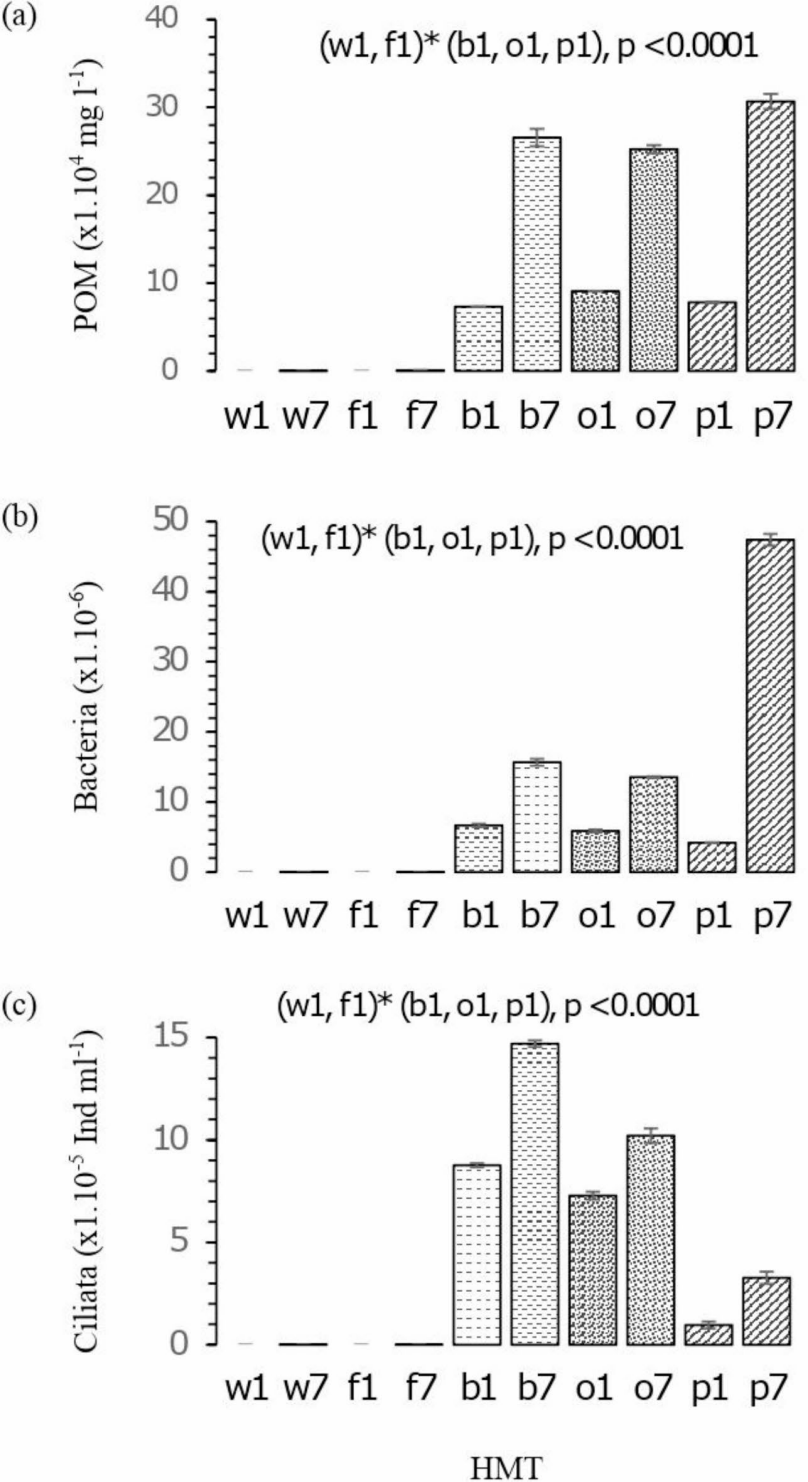
POM (**a**), bacteria (**b**) and ciliate (**c**) densities. w: CSw; f: CSf; b: HMTb; o: HMTo; p: HMTp; w1to p1: Observations on the day 1(D_1_); w7 to p7: Observations on the day 7 (D_7_).

As of incubation onset on D_1_, the HMT biological analysis results revealed (Fig. 2) the presence of total germs (bacteria), ciliates (protozoa) and particulate organic matter (POM). For the latter, from a quantitative standpoint, a highly significant difference between HMT and CS was observed at D_1_ (*p* < 0.0001; Fig. 2a). Note that the CS samples were free of POM, bacteria and ciliates, which means that the origin of the latter was closely dependent on the nature of the manure used in the HMT preparation and the hosted microbiota population. Several authors have noted that bacteria, protozoa, fungi and archaea inhabiting farm compost and involved in organic matter (i.e. lignocellulose) degradation derive from livestock guts and from the farming environment where the manure is found, while the quality, quantity and diversity of these organisms closely depends on the materials used in the degradation or composting process, and the organic matter quality in the finished compost^95–97^. Significant increases in POM content (Fig. 2a), as well as bacterial (Fig. 2b) and ciliate (Fig. 2c) densities were observed between D_1_ and D_7_ (*p* < 0.0001) in all three HMT. If we juxtapose our findings with those from studies on aqueous media such as wastewater, the increase in POM, which serves as a carbon and nitrogen reservoir, would be closely dependent on their hydrolytic degradation rate, which in turn depends on the quality and quantity of microbiota present on site. Ciliated protozoa are especially involved in controlling these dynamics, partly via POM and bacteria phagocytosis. The latter are involved in POM degradation by dissolved organic matter assimilation^44–46,98–101^. The Pearson correlation matrix (Table 2) revealed a very close correlation between POM and bacteria. Conversely, a negative correlation was noted between the ciliate-bacteria (r =− 0.94) and ciliate-POM (r =− 0.81) ratios. We suggest that the high ciliate densities observed in HMTb and HMTo (Fig. 2c) were related to the high POM and bacteria concentrations, with the latter having served as ciliate prey, thereby explaining their decrease on D_7_ (Fig. 2). For HMTp, the lack of ciliate development likely enabled the proliferation of POM-consuming bacteria. Previous research has indicated that POM is slowly biodegradable^44,47,102^. HMTb and HMTo POM was hence likely controlled by ciliated protozoa in the same way as bacteria. In CS, minor bacteria, ciliate and POM concentrations were documented due to the absence of organic matter and to the incubation effect that, in the presence of light and the low nutrient contents in the water, especially nitrogen, allowed their development. This microbiota development intensity in HMT could be favoured by the physicochemical properties of HMT, thereby allowing dissemination of hosted microorganisms, particularly bacteria and ciliates whose activity and multiplication could be enhanced by the fermentation process that occurs during incubation^5,103,104^.

**Table 2:** Correlations between POM, bacteria and ciliates compounds according to Pearson correlation matrix tests.

Variables	POM	Ciliata	Bacteria
POM	1		
Ciliata	− 0.810	1	
Bacteria	0.932	− 0.939	1

POM: Particulate organic matter.

## Conclusions

This study reveals key hydrobiological characteristics of manure-based homemade tea (HMT) and the factors that influence their nutrient profiles and microbial dynamics, particularly when prepared without agitation. The rapid progression to anoxic and then anaerobic conditions, especially in poultry manure tea (HMTp), highlights how manure type and incubation duration shape oxygen consumption, microbial activity, and nutrient solubility, which directly affect HMT bioactive properties.

High nitrogen compound levels across all HMT formulations underscore their potential as effective biofertilizers. HMTp presented notable nitrogen advantages, suggesting it could be especially beneficial for nitrogen-demanding crops. Our results suggest that processes like anammox and microaerobiosis may occur under anaerobic conditions, potentially contributing to nitrogen loss through denitrification. Reducing the incubation period to four days may help curb nitrogen volatilization, preserving more bioavailable nitrogen and facilitating manure reuse in successive batches, thus optimizing resource efficiency and preparation time.

From an agronomic standpoint, HMT presents a sustainable alternative to synthetic fertilizers, supporting reduced reliance on chemical inputs and promoting environmentally responsible farming practices. To enhance nutrient management, future research should quantify nitrogen balance in HMT, identifying specific loss pathways and refining nitrogen retention strategies. Additionally, further exploration of HMT bound ciliates and particulate organic matter (POM) could shed light on their roles in plant health and nutrient cycling, enabling targeted applications in crop systems.

Though this study focused on nitrogen dynamics, phosphorus and potassium, as a key nutrient for plant growth and microbial functions, should be investigated in future studies. These fi2ndings lay a foundation for improving HMT production techniques to maximize agronomic benefits, minimize environmental impacts, and support a transition toward eco-friendly, resource-efficient agricultural systems.

## Data Availability

The data that support the findings of this study are available from the corresponding author upon reasonable request.
